# SAVSDN: A Scene-Aware Video Spark Detection Network for Aero Engine Intelligent Test

**DOI:** 10.3390/s21134453

**Published:** 2021-06-29

**Authors:** Jie Kou, Xinman Zhang, Yuxuan Huang, Cong Zhang

**Affiliations:** 1 School of Electronic and Information Engineering, MOE Key Lab for Intelligent Networks and Network Security, Xi’an Jiaotong University, Xi’an 710049, China; kj317099295@stu.xjtu.edu.cn (J.K.); zhangxinman@mail.xjtu.edu.cn (X.Z.); hyx0903@stu.xjtu.edu.cn (Y.H.); 2 AECC Sichuan Gas Turbine Establishment, Mianyang 621000, China

**Keywords:** video object detection, small object detection, spark detection, video codec, deep ConvLSTM network, aero engine intelligent test

## Abstract

Due to carbon deposits, lean flames, or damaged metal parts, sparks can occur in aero engine chambers. At present, the detection of such sparks deeply depends on laborious manual work. Considering that interference has the same features as sparks, almost all existing object detectors cannot replace humans in carrying out high-precision spark detection. In this paper, we propose a scene-aware spark detection network, consisting of an information fusion-based cascading video codec-image object detector structure, which we name SAVSDN. Unlike video object detectors utilizing candidate boxes from adjacent frames to assist in the current prediction, we find that efforts should be made to extract the spatio-temporal features of adjacent frames to reduce over-detection. Visualization experiments show that SAVSDN can learn the difference in spatio-temporal features between sparks and interference. To solve the problem of a lack of aero engine anomalous spark data, we introduce a method to generate simulated spark images based on the Gaussian function. In addition, we publish the first simulated aero engine spark data set, which we name SAES. In our experiments, SAVSDN far outperformed state-of-the-art detection models for spark detection in terms of five metrics.

## 1. Introduction

During aero engine testing, the engine frequently starts and stops with a high rotation speed at high temperature and pressure for a long time [1]. Under these conditions, the phenomenon of spurting anomalous sparks may occur due to carbon deposits with low thermal conduction [2], lean flames [3], or damaged metal parts burning in the combustion chamber [4]. However, undesirable carbon deposits may deactivate the catalyst, block the reactor tube, and lead to a pressure drop [5]. Moreover, lean flames are susceptible to incomplete mixing and local equivalence ratio exceeding the overall lean equivalence ratio, which could lead to hot spots and an increase in emission particulate matter [3]. The damaged metal parts may be the result of compressor fouling and turbine erosion, which are considered as the main causes of engine performance degradation [6]. Therefore, abnormal sparks provide evidence of the instability of performance, structure, and operation of aero engines, which need to be detected in time to prevent huge economic losses.

Recently, non-intrusive measurement techniques have become more popular than conventional extractive techniques because they do not require a sampling rake in the exhaust plume [7]. For example, the flame image taken by a visible light high-speed camera was used to analyze the spatio-temporal characterization of the flame dynamics and combustion oscillations [3]. Lowe et al. [8] applied laser techniques to measure the cross-sectional distribution of carbon dioxide in an aero engine exhaust plume without introducing any disturbance to exhaust flow. Powrie et al. [4] applied the electrostatic sensor to sense the electrostatic charge associated with debris present in the gas path of jet engines or gas turbines. However, the electrostatic sensor had poor anti-interference ability and could not sense sparks caused by carbon deposits. There is currently no independent and complete intelligent monitoring system for spark detection which largely relies on human participation. Furthermore, such manual detection may produce many missed detections, resulting in the monitoring results being mixed with artificial error. Therefore, better intelligent analysis systems are urgently needed to link the engine visible light image data to its health status which could liberate human resources and realize the intelligent detection of anomalous sparks in aero engine testing surveillance.

**Figure 1:**
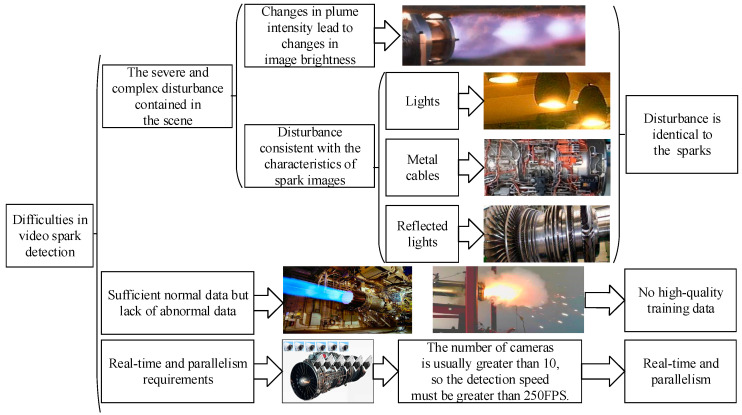
The three main difficulties in video spark intelligent detection.

Figure 1 shows the three difficulties faced by intelligent spark detection. First, the feature of a single spark is a bright line segment, which is completely consistent with the features of the lights, metal cables, and reflected light in the image. Second, a lack of abnormal spark data hinders the training of supervised learning models. Third, more than 10 cameras are used to monitor the status of aero engines, which requires the spark detection algorithm to be real-time and parallel.

Traditional algorithms for object detection usually include scale-invariant feature transform (SIFT) [9,10,11], histogram of gradient (HOG) [12,13], and so on. Daoud et al. [14] have proposed a fully pipelined hardware accelerator architecture for SIFT keypoint descriptor matching. Bakr et al. [15] presented a method for action recognition by using the histogram of oriented depth gradients. Deep learning has gradually taken the place of traditional algorithms in the field of object detection, and many scholars have researched image object detection based on deep learning techniques [16,17,18]. Jiang et al. [19] improved the performance of You Only Look Once v4-tiny (YOLOv4-tiny), which is more suitable for real-time object detection, especially for development on embedded devices. Ren et al. [20] added a region proposal network (RPN) to the faster RCNN model [21] and achieved state-of-the-art object detection accuracy at that time. However, we found that the existing methods in the field of object detection cannot satisfactorily perform spark detection. We boiled this down to two important reasons. First, the sparks have simple morphological features, appearing as bright line segments in surveillance videos. However, many objects in the chamber that are present as interference bright line segments have the same morphological features as sparks. This leads to over-detection in the object detectors which use a single image as input. Second, affected by the high-speed airflow in the aero engine, the objects with bright line segment features are subject to complex movements. As it is hard to artificially design spatio-temporal features with good discrimination, high quality, and stability [18], the traditional algorithms cannot effectively distinguish sparks from interference bright line segments in the spatio-temporal domain.

Many video object detection algorithms have arisen with the proposal of ConvLSTM [22], which outperforms FC-LSTM and captures spatio-temporal correlations better by extending the fully connected LSTM to have convolutional structures in its transitions. Compared with traditional algorithms and image object detection, video object detection can obtain temporal context information, which can be used to remove the bottlenecks inherent to the previous technologies [23,24,25,26]. Therefore, video object detection has enjoyed great popularity in the field of object detection [27,28,29]. Video object detection can be mainly categorized into high-precision approaches [30,31,32,33] and high-speed approaches [34,35,36,37]. A high-speed approach makes full use of the temporal context to speed up the process of detection, while high-precision approaches improve the performance by focusing on the temporal context [25]. Related models proposed in recent years include FGFA [31], D&T [33], MEGA [32], LSTS [34], and SELSA [30]. We found, through experiments, that the state-of-the-art video object detector, MEGA, is also unable to perfectly detect sparks, possibly because it focuses on using the candidate boxes of adjacent frames to guide the current detection. Eventually, over-detection error on each image will still gradually accumulate, deteriorating the following detection results.

For spark detection, over-detection (rather than missed detection) often occurs. We assume that the over-detection results are due to the features similar to sparks and interference bright line segments within a single frame. We believed that the lack of spatio-temporal information leads to spark over-detection. Due to the high speed of sparks, we found that the same spark will be captured at different positions in two or three adjacent frames under the premise of 25 fps video. On the contrary, interference bright line segments either do not move or move slowly, almost appearing at a fixed position in 10 or more adjacent frames. So, there are significant spatio-temporal feature differences between sparks and bright line segments in the spatio-temporal domain. The detectors must make use of this spatio-temporal context information contained in the surveillance videos to accurately sense the two kinds of bright line segment feature in the scene, thus fusing these two kinds of information and improving the accuracy of anomalous spark detection. The pioneering LSTM encoder–decoder framework proposed in [38] provides a general framework for sequence-to-sequence learning problems by training temporally concatenated LSTMs: one for the input sequence and another for the output sequence. Shi et al. [22] first proposed the ConvLSTM encoder–decoder framework to make up for the lack of LSTM spatial expression capabilities, which led to an inability to effectively extract spatio-temporal features in image sequences. Inspired by the ConvLSTM encoder–decoder framework, we propose a cascade model, consisting of a video codec and an image object detector for anomalous spark detection. To distinguish sparks from the interference bright line segments, the codec extracts a deep spatio-temporal feature image from the video, which is used as the input to the image object detector.

A sequence-to-sequence learning model can compress and encode long temporal series data information into a fixed-length vector representation, based on a deep learning model; then, another deep learning model can decode the vector as the predictive output [38]. We improve the sequence-to-sequence learning model to make it more suitable for learning the difference between disturbing bright line segments and sparks, and we propose an information fusion mechanism to fuse these two kinds of information. By dividing the input into long and short sequences, ConvLSTM learns long-term features from the long sequence, while another ConvLSTM learns short-term features from the short sequence. A ConvLSTM fuses these two kinds of information and decodes it as the input of the image object detector. By learning the conditional distribution of a code image generated by several successive image sequences, it can encode the code image into a deep temporal and spatial characteristic, which can distinguish sparks from interfering bright lines. By cascading the ConvLSTM structure with an image object detector, the detector is endowed with the ability to obtain spatio-temporal context information so that it can extract the spatio-temporal non-linear correlations and the long spatio-temporal deep dependency; our experiments showed that we could reduce the over-detection successfully. In addition, considering the lack of aero engine spark data sets and the limited spark morphological features generated when using a simulated aero engine, it is difficult to train a spark detection model with excellent performance. We attempted to use real anomalous spark data to train our model, which did not work very well. Inspired by a generation algorithm for raindrop appearance [39], we added spark images generated by the Gaussian function to the normal aero engine data to solve the problem. Thus, we could use the normal aero engine data to generate a large amount of training data. Our main research contributions are as follows:

1. We propose a cascade model, consisting of a video codec and an image object detector for anomalous spark detection in aero engines. The codec, which extracts a deep spatio-temporal feature image that can distinguish sparks from interference bright line segments, is based on ConvLSTM sequence-to-sequence learning and an information fusion mechanism;
2. To solve the lack of anomalous spark data, we propose a method for generating high-quality training data. Specifically, we add spark images generated by the Gaussian function to normal aero engine data;
3. We establish a SAES data set, which is the only publicly published data set in the field of spark detection. The images of the data set are all derived from the simulated aero engine chamber which contains ample interference. SAES is the best choice to test the performance of spark detection models at present.

The remainder of the paper is organized as follows. Section 2 details the materials and method of detecting anomalous sparks. Section 3 introduces the SAES data set, the method of training data generation, and the experiments. Finally, Section 4 delivers our conclusions.

## 2. Materials and Methods

For spark detection, we improved the existing sequence-to-sequence learning model [40] to make it more suitable for learning the difference between disturbing bright line segments and sparks, and we proposed an information fusion mechanism to fuse the two kinds of information. In this section, we first describe the classical sequence-to-sequence model and ConvLSTM. Then, we detailly describe the framework of our designed SAVSDN and explain the execution process of spark detection.

### 2.1. Sequence-to-Sequence Model and ConvLSTM

The classical sequence-to-sequence learning model can compress and encode long temporal sequence data into a fixed-length vector, based on the use of a deep learning model; then, another deep learning model can decode the vector into the predictive output. The sequence-to-sequence model is a general learning framework [40]. Then, the feature image is sent to an object detector for the further detection of anomalous sparks. The computational process of the classical sequence-to-sequence learning model can be described as follows:

(1) $$\begin{array}{l} P\left(f_{t+1},f_{t+2},\ldots ,f_{t+p}|X_{t-l+1},X_{t-l+2},\ldots ,X_{t}\right)\\ =P(f_{t+1}|c_{t})\cdot P(f_{t+2}|c_{t},f_{t+1})\cdot P(f_{t+3}|c_{t},f_{t+1},f_{t+2})\\ \cdot \ldots \cdot P(f_{t+p}|c_{t},f_{t+1},f_{t+2},\ldots ,f_{t+p-1})\\ =\prod_{k=1}^{p}P(f_{t+k}|c_{t},f_{t+1},f_{t+2},\ldots ,f_{t+k-1}) \end{array}$$

where $\left(X_{t-l+1},\ldots ,X_{t}\right)$ is an input sequence and $\left(f_{t+1},\ldots ,f_{t+p}\right)$ is its corresponding output sequence whose length p may differ from l. p(the Prediction Size) is the number of multi-step predictions, and the size of the window for the input temporal sequences data is l (the lookup size).

**Figure 2:**
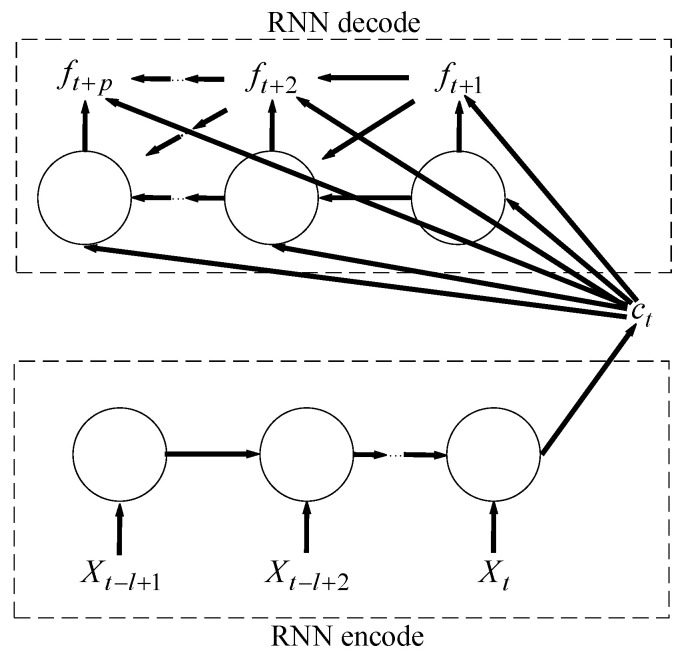
The sequence-to-sequence model.

In fact, the classical sequence-to-sequence model is widely used in machine translation. The goal of the model is to estimate the conditional probability $p(f_{t+1},\ldots ,f_{t+p}|x_{t-1+1},\ldots ,x_{t})$. The encoder computes this conditional probability by first obtaining the fixed dimensional representation c of the input sequence given by the last hidden state of the RNN, and then the decoder computes the probability of $y_{1},\ldots ,y_{T^{\prime }}$ with a standard formulation whose initial hidden state is set to the representation c of $x_{1},\ldots ,x_{T}$. When the decoder decodes the encoding result of an input sequence of one language into an output sequence of another language, RNN as a decoder will output $f_{t+1},f_{t+2},\ldots ,f_{t+p}$ sequentially one by one, and the predicted output $f_{t+i}$ in each step should be based on the field dimensional representation c and the previously predicted output $f_{t+1},f_{t+2},\ldots ,f_{t+i-1}$ to calculate. This sequence-to-sequence model is shown in Figure 2.

A recurrent neural network (RNN) is usually used for codecs based on sequence-to-sequence learning models [38]. As an RNN takes sequence data as input and recurses the input in the order in which the data are entered, its memorability, parameter sharing, and turing completeness can be used to learn the non-linear features of input sequences. For the input of temporal sequences, at each time step *t*, the output of the RNN hidden state is computed as follows:

(2) $$h_{t}=RNN(h_{t-1},x_{t})$$

RNN, as the encoder, reads each time step datum of the temporal sequence sequentially, and updates the hidden state of the RNN, according to Equation (2). After reading to the end of the sequence, we take the hidden state of the RNN as the encoder’s coding result, c. As a decoder, an RNN network with a given hidden state predicts the value of the next time step and outputs the value as the prediction result of the model. In particular, the decoder fuses the previous prediction output with the current input in each prediction step. Therefore, at time step *t*, the hidden state of the decoder can be computed as follows:

(3) $$P(f_{t}|f_{t-1},f_{t-2},\ldots ,f_{1},c)=RNNdec(h_{t},f_{t-1},c)$$

The hidden state of RNN can guide the next prediction. Although, in theory, RNN is perfectly capable of capturing long-time dependency, it was proved that RNN is almost impossible to learning long sentences on machine translation tasks. To solve the long-time dependency problem, LSTM, a special RNN, replaces the simple structures in standard RNN, such as the tanh layer, with three gating units. The focus of LSTM is cell state, with only some linear relationships that ensure that information will be passed down without changing. In addition, LSTM can depend on a gate structure consisting of the sigmoid neural layer and a dot product unit to delete or add some of the information. The output of the sigmoid neural layer is between 0 and 1. The output of 0 means that the information cannot pass at all, and the output of 1 means that all the information passes. The main drawback of LSTM is that it is impossible to encode spatial information well. In order to successfully model the spatio-temporal relationships, Shi proposed ConvLSTM which applies convolutional structures in both the input-to-state and state-to-state transitions. This is shown in the following formula:

(4) $$i_{t}=\sigma (W_{xi}*X_{t}+W_{hi}*H_{t-1}+W_{ci}\circ C_{t-1}+b_{i})$$

(5) $$f_{t}=\sigma (W_{xf}*X_{t}+W_{hf}*H_{t-1}+W_{cf}\circ C_{t-1}+b_{f})$$

(6) $$C_{t}=f_{t}\circ C_{t-1}+i_{t}\circ \tanh(W_{xc}*X_{t}+W_{hc}*H_{t-1}+b_{c})$$

(7) $$o_{t}=\sigma (W_{xo}*X_{t}+W_{ho}*H_{t-1}+W_{co}\circ C_{t}+b_{o})$$

(8) $$H_{t}=o_{t}\circ \tanh(C_{t})$$

where ∗ represents the convolution operator, ∘ denotes the Hadamard product, $X_{1},X_{2},\ldots ,X_{t}$ are the whole inputs, $C_{1},C_{2},\ldots ,C_{t}$ indicate the states of the cell, $H_{1},H_{2},\ldots ,H_{t}$ show the hidden states, and the gate $i_{t},f_{t},o_{t}$ is a three-dimensional tensor. It can be also simplified as:

(9) $$H_{t},C_{t}=ConvLSTM(X_{t},H_{t-1},C_{t-1})$$

We believed that, by learning the conditional distribution of an image which is encoded from several successive image sequences, ConvLSTM can encode successive image sequences into a deep spatio-temporal feature image that can distinguish sparks from interference bright line segments. In practical applications, we only needed to encode several successive image sequences to obtain deep spatio-temporal features image that can distinguish sparks from interference bright line segments. Our specific structure is described in the following passage. The computational process can be simplified as follows:

(10) $$P(f|X_{t-8},X_{t-7},\ldots ,X_{t})=P(f|c_{t})$$

### 2.2. Overview of Cascading Video Codec

**Figure 3:**
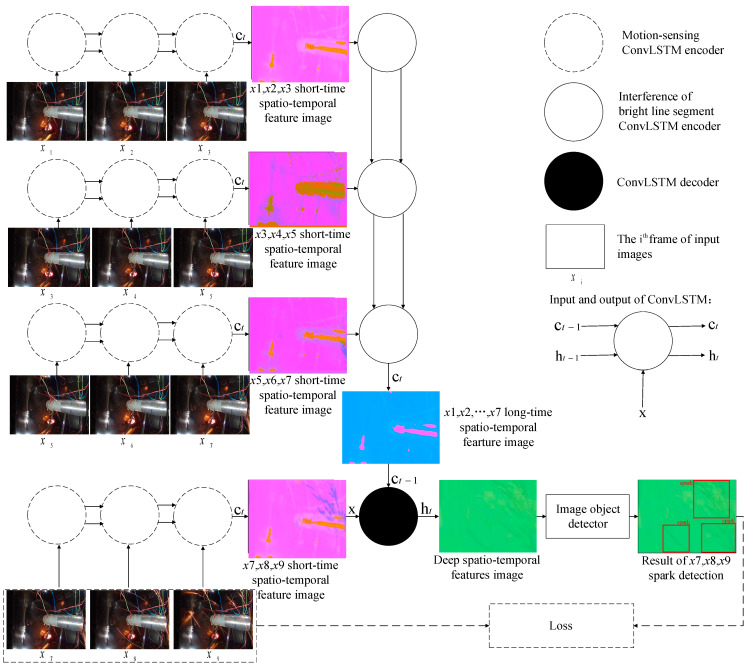
The information fusion-based spark detection model.

For the respective spatio-temporal feature learning of sparks and interference bright line segments, we needed to extract the motion features of all relevant bright line segments in the spatio-temporal domain and recognize which ones belong to interference bright line segments in the scene. In this section, we detail our spark detection method. We first introduced the outline of our video codec-based spark detection model. Then, we showed how to take into account the spatio-temporal correlations and long dependency of different time step lengths. The outline is shown in Figure 3.

Figure 3 illustrates the framework of our proposed aero engine spark detection model. The model does not follow the pattern of popular video object detectors, which generally make use of candidate boxes from adjacent frames to speed up the current frame detection, or to make up for the missing features of the object in the current frame due to motion blur, vibration of cameras, and so on in order to reduce missed detections [32]. Differing from such video object detection methods, each result of our model was based on the spatio-temporal features extracted from the spatio-temporal context sequences, rather than candidate boxes of adjacent frames. There were two main stages. First, the spatio-temporal sequences were encoded and decoded to obtain feature images. Second, spark features in the feature images were detected by the object detector. In the first step, we used the codec to encode and decode the image sequences and to obtain feature images that can distinguish sparks from the interference bright line segments. In the second step, the object detector obtained the position of sparks through the detected spark features in the feature images.

Motivated by the success of sequence-to-sequence learning algorithms, recent machine translation methods follow the pattern of sequence-to-sequence learning [41]. They aim to extract temporal correlation features from input sequences, then decode these compressed features in the form of sequences. Inspired by this, for spark detection, the codec needs to learn a conditional distribution of prediction sequences with a fixed-length sequence as the condition, and then encode and decode the image sequences of the video to obtain a high-level spatio-temporal neural feature image which can help to distinguish sparks from the interference bright line segments. The process of encoding and decoding image sequences is as follows:

(11) $$\begin{array}{l} \tilde{Y}=\underset{Y}{\arg\max}P(Y|{\hat{X}}_{t-l+1},{\hat{X}}_{t-l+2},\ldots ,{\hat{X}}_{t})\\ \approx \underset{Y}{\arg\max}P(Y|E_{encoder}({\hat{X}}_{t-l+1},{\hat{X}}_{t-l+2},\ldots ,{\hat{X}}_{t}))\\ \approx D_{forecaster}(E_{encoder}({\hat{X}}_{t-l+1},{\hat{X}}_{t-l+2},\ldots ,{\hat{X}}_{t})) \end{array}$$

where $\left({\hat{X}}_{t-l+1},{\hat{X}}_{t-l+2},\ldots ,{\hat{X}}_{t}\right)$ is the input image sequence of the codec, and $\tilde{Y}$ is the result of the codec. In particular, l=9 in SAVSDN.

Here, argmax is a kind of function which attempts to find the corresponding parameter set Y when the maximum value of the conditional probability $P\left(Y|X_{t-l+1},X_{t-l+2},\ldots ,X_{t}\right)$ is obtained. $E_{encoder}$ represents the encoding function. $D_{forecater}$ represents the decoding function. The encoder first encodes the input image sequence into a feature vector $E_{encoder}(X_{t-l+1},X_{t-l+2},\ldots ,X_{t})$. Then, the decoder decodes the vector and we will obtain an output feature image $Y=D_{forecater}(E_{encoder}(X_{t-l+1},X_{t-l+2},\ldots ,X_{t}))$. Y is the parameter set when the maximum value of the conditional probability $P\left(Y|X_{t-l+1},X_{t-l+2},\ldots ,X_{t}\right)$ is obtained.

The codec design needed to consider a key point: there are significant spatio-temporal feature differences between sparks and bright line segments in the spatio-temporal domain, under the premise of the 25 fps video. Due to the high speed of sparks, the same spark is captured at different positions in two or three adjacent frames. On the contrary, interference bright line segments do not move or move slowly, almost appearing at a fixed position in 10 or more adjacent frames.

The encoders and decoders are always based on RNNs. Nevertheless, the deep features obtained from encoding the previous input image sequences may be diluted by the subsequent input encoding features. If sparks appear at the beginning of long sequences, the extracted deep features may not contain spark non-linear correlation features [41], thus not eliminating over-detection.

In addition, we noticed that the same spark will be captured at different positions in two or three adjacent frames under the premise of the 25 fps video due to the high speed of sparks, while the interference bright line segments do not move or move slowly, almost appearing at a fixed position in 10 or more adjacent frames. We applied ConvLSTM to exploit this spatio-temporal features in the input sequences. Next, we divided every nine frames into the first seven frames and the last three frames. In the first seven frames, global scene features and long-range features were extracted, while the correlated non-linear spatio-temporal features were extracted from the last three frames; then, ConvLSTM was used to fuse all the features and decode them to obtain a feature image. The first seven frames were only used to extract the non-spark-related global scene features and long-stage features, enabling the object detector to distinguish sparks from interference bright line segments. Moreover, we used a parameter-sharing network to extract spatio-temporal information from the whole input sequences. The parameter-sharing network enabled the consistency of the extracted features and reduced over-detection. Finally, our model learned the spatio-temporal differences between sparks and the interference bright line segments from spatio-temporal sequences. In the classic sequence-to-sequence model for machine translation, the decoder and encoder usually choose the same model component, such as RNN. The difference is that, in order to enable the video codec to extract the feature image that can distinguish the spark from the interference bright line segments, the codecs in SAVSDN do not adopt the same model component, but adopt a design based on the information fusion mechanism. The specific structure is described in Section 2.3. In the ablation experiment in Section 3.4, it is also proved that SAVSDN has a better performance in sparks detection compared with the classical sequence-to-sequence models.

### 2.3. Execution Process

First, we obtained nine adjacent frames from the 25 fps aero engine surveillance video. Then, all nine images were scaled to 1024 by 1024 pixels. After a process of image graying, we obtained an input $X=\{x_{1},x_{2},\ldots ,x_{9}\}$, of shape 9×H×W×C. In our experiment, H=1024, W=1024 and C=3. In order to match human brightness perception, we used a weighted combination of the RGB channels as follows:

(12) Gray=0.3R+0.59G+0.11B

where R, G and B represent linear red, green, and blue channels. The Gray represents the output of this grayscale algorithm.

The reason for the image graying here is that the sparks produced by metal combustion have a wide color gamut. Data-driven deep learning models require massive amounts of training data to learn the complex and varied color features of sparks. This is unrealistic, due to the lack of aero engine abnormal spark data sets. The graying operation allowed the model to focus only on the bright line segment morphological features in the scene, instead of the complex and varied color features. Next, X was divided into four groups of image sub-sequences: frames 1–3, $X_{1}=\{x_{1},x_{2},x_{3}\}$; frames 3–5, $X_{2}=\{x_{3},x_{4},x_{5}\}$; frames 5–7, $X_{3}=\{x_{5},x_{6},x_{7}\}$; and frames 7–9, $X_{4}=\{x_{7},x_{8},x_{9}\}$. The shape of each sub-sequence was 3×H×W×C. The codec result can be expressed as Y={f}.

#### 2.3.1. Motion Encoding Model

Then, the four sub-sequences were encoded, respectively, using the motion-sensing encoder composed of ConvLSTM. Extracting the deep spatio-temporal features from each sub-sequence, we obtained four spatio-temporal context vectors with the shape $1\times H\times W\times C^{\prime }$. Here, $C^{\prime }=8$ in order to extract more features. There were four groups of weight-sharing ConvLSTM in SAVSDN, which were used to capture motion features of image sub-sequences, respectively, as shown in the following formula:

(13) $$h_{i,t},c_{i,t}=ConvLSTM(x_{i,t},h_{i,t-1},c_{i,t-1}),1\leq i\leq 4,1\leq t\leq 3$$

where $x_{i,t}$ are the data of $x_{i}$ in the $t^{\mathrm{th}}$ time step, $c_{i,t}$ is the motion-sensing code of each image sub-sequence at time step t, and $h_{t}$ and $c_{t}$ are the hidden state and the hidden context vector of the motion-sensing encoder, respectively, in the $t^{\mathrm{th}}$ time step $x_{i,t}$ will be used by the $i^{th}$ ConvLSTM unit to capture the motion features for the prediction of feature image $c_{i,t}$ in $t^{\mathrm{th}}$ time step. The above equation can be simplified as follows:

(14) $$H_{motioni},C_{motioni}=Enc_{motion}(X_{i}),\;\;\;1\leq i\leq 4,\;\;\;1\leq t\leq 3$$

where $C_{motioni}$ represents the last predicted cell state $c_{i,3}$, which includes motion features of three image sequences. $H_{motioni}$ represents the last predicted output $h_{i,3}$. $X_{i}$ is the $i^{th}$ image sub-sequence.

**Figure 4:**
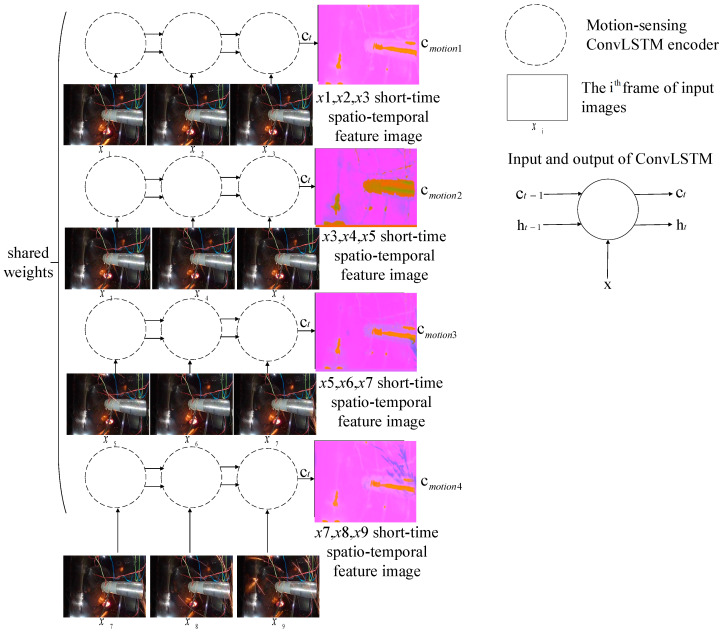
The proposed motion encoding model.

We tried to use independent encoders, resulting in poor performance. So, we designed a parameter-sharing encoder to encode each sub-sequence, ensuring that we could exploit the same spatio-temporal features. Furthermore, as $c_{i,t}$ can better represent long dependency features and spatial correlation features, we chose $c_{i,t}$ as the hidden context vector, rather than $h_{i,t}$. The proposed motion encoding model is shown in Figure 4.

#### 2.3.2. Scene Encoding Model

To avoid the spatio-temporal features being diluted by subsequent inputs when ConvLSTM extracts long image sequences, and to obtain deep spatio-temporal feature image of long-existing interference bright line segments in the first seven frames, we spliced the hidden context vectors encoded from $X_{1}$, $X_{2}$, and $X_{3}$ into a new spatio-temporal sequence, $C=\{c_{motion1},c_{motion2},c_{motion3}\}$, with the shape $3\times H\times W\times C^{\prime }$. Then, C is sent to another ConvLSTM-based long-range global scene encoder for encoding to obtain the long-term global scene features $h_{mask}$. As the interference bright line segments are generated by the inner structure of the aero engine chamber, many spatio-temporal non-linear correlations exist in C, which can be encoded into $c_{mask}$:

(15) $$h_{mask},c_{mask}=Enc_{scene}(C)$$

where $c_{mask}$ is the cell state predicted by ConvLSTM in the last step, which is the encoding results of three motion feature images and includes features of long-time existing interference. $h_{mask}$ is the output predicted by ConvLSTM in the last step. C represents a new image sequence composed of three motion feature images.

**Figure 5:**
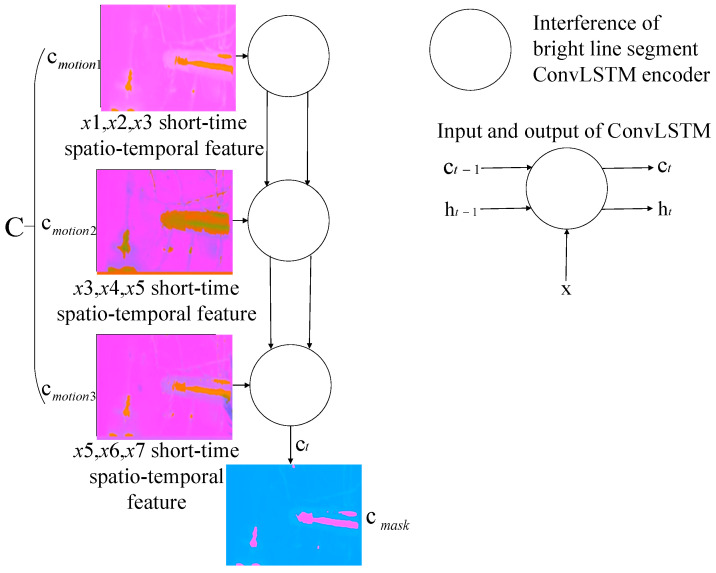
The proposed scene encoding model.

By grouping and encoding the input sequences, we acquired global scene features and long-range features from the first seven frames and obtained non-linear spatio-temporal correlation features of sparks. The proposed scene encoding model is shown in Figure 5.

#### 2.3.3. Decoding Model Based on an Information Fusing Mechanism for Spark Detection

The other ConvLSTM-based decoder, taking $c_{mask}$ as the long-range hidden state, decoded the motion-sensing hidden context vectors of sparks and interference bright line segments in $X_{4}$. Through fusing the two kinds of information, the decoder outputted non-linear spatio-temporal correlation features f, which could be used to distinguish sparks from interference bright line segments. The decoding process can be expressed as:

(16) $$f=ConvLSTM(c_{motion4},\vec{0},c_{mask})$$

where the feature image f, with shape 1×H×W×C, is input to the subsequent object detector, helping to screen all the spark features in $X_{7}$, $X_{8}$, and $X_{9}$.

**Figure 6:**
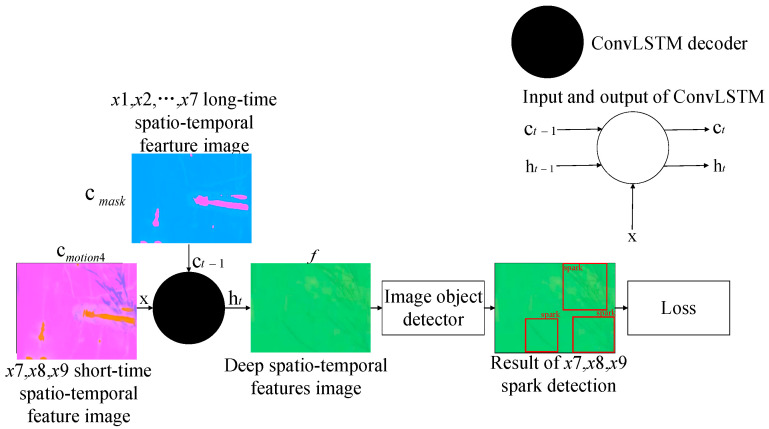
The proposed decoding model based on an information fusing mechanism.

In our experiment, C=3 and $C^{\prime }=8$. The kernel size of ConvLSTM was 3. We chose YOLOv5 [42] as the object detector. SAVSDN was based on end-to-end deep learning. The loss function follows the original loss function of the object detector, and was calculated from the output of the object detector, $x_{7}$, $x_{8}$, and $x_{9}$, as well as the categories and positions of the sparks and the true values. The proposed decoding model for spark detection is shown in Figure 6.

**Figure 7:**
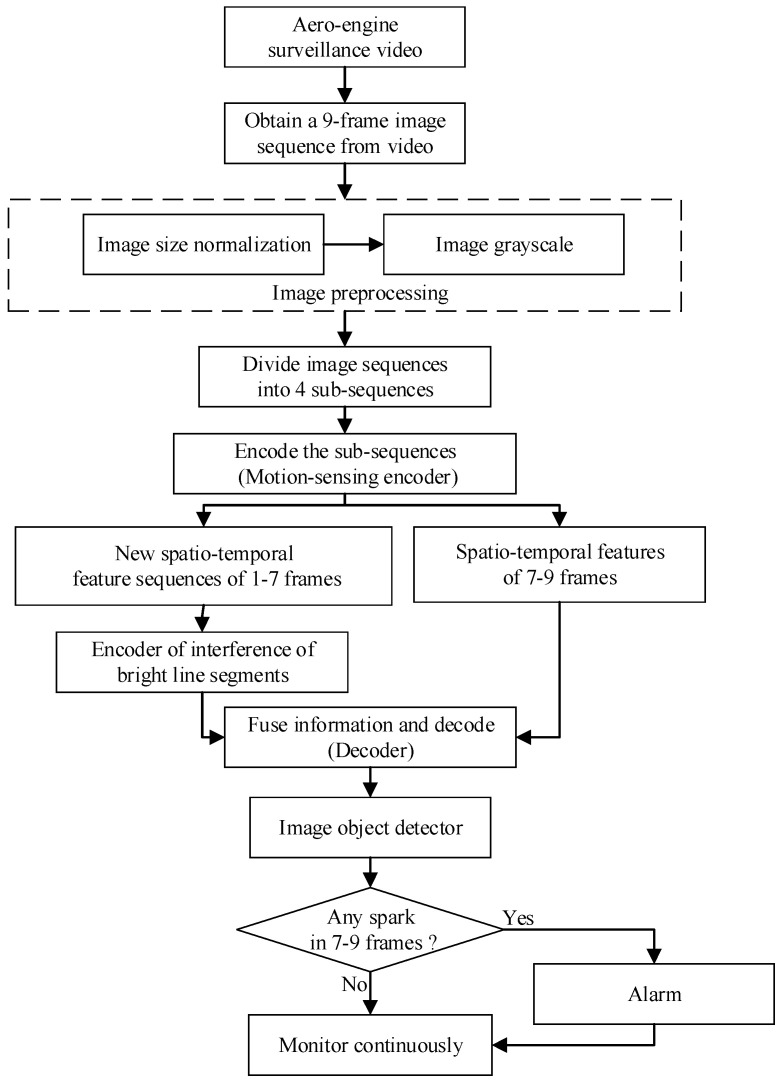
Flow chart of spark detection.

The flow chart of spark detection is shown in Figure 7.

In fact, we proposed a real-time anomalous spark video detection method based on deep learning for aero engine testing. Each time, the input was a sequence of nine consecutive frames, and the output was the position of all the anomalous sparks in the last three frames. In particular, the first seven frames were used by SAVSDN to perceive the bright line segments interference in the scene, so that the network could easily distinguish the sparks and the interference. First of all, nine frames of image sequences were inputted for preprocessing, including image size normalization and image grayscale. Then, the input was divided into 4 groups of image sub-sequences in chronological order, and each group of image sub-sequence contained 3 consecutive images. Next, four weight-sharing ConvLSTM were used to extract motion feature images from the four image sub-sequences, respectively. Moreover, a new sequence will be created by integrating the feature images corresponding to the first three groups of image sub-sequences. In addition, the new sequence will be sent to another ConvLSTM to extract scene perception feature images, which contains long time existing interference of bright line segments. Moreover, after obtaining scene perception feature images, another ConvLSTM was used to integrate the scene perception feature images and the motion feature images corresponding to the last image sub-sequence, and was then decoded to obtain a spatio-temporal deep feature image. The spatio-temporal deep feature image contained the spatio-temporal features that could distinguish sparks from the interference of bright line segments, which will be sent to the subsequent image object detector to detect spark features again. If the image object detector detects a spark, the human-computer interaction terminal will alarm and continuously monitor, otherwise continue to monitor.

## 3. Experiments

In this section, we introduce the first simulated aero engine spark data set, which we have published. The data set contains complex and strong interference, which makes it extremely challenging to conduct spark detection using it. We tested the spark detection ability of our model on this data set to demonstrate its superior spark detection performance. Secondly, to solve the problem of a lack of real aero engine anomalous spark data sets, contributing to the inability to train a high-precision, robust, and generalizable spark detection model, we proposed a method to obtain high-quality training data. The simulated spark images, which could be added to the normal aero engine image, were generated using the Gaussian function. As there are multiple possible reasonable labels for multiple sparks, we could not use the average precision (AP) as the evaluation metric. Therefore, we proposed new metrics for spark detection performance evaluation. Then, we performed a number of ablation studies in order to fully understand the elements and effects of SAVSDN. Finally, we also compared the proposed model with state-of-the-art object detectors and video object detectors in order to show the unique effectiveness and generality of our model.

### 3.1. SAES Data Set for Model Testing

**Figure 8:**
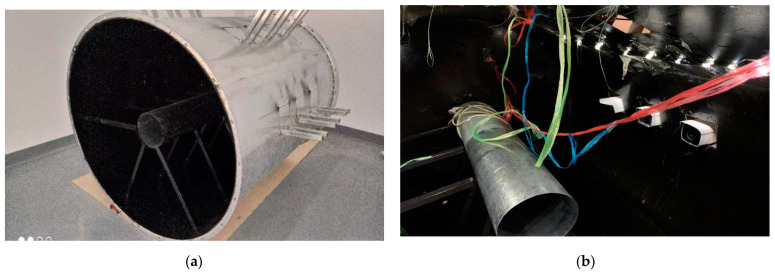
Data collection scene used for SAES: (**a**) Simulated aero engine chamber, which contains multiple camera positions, a simulated aero engine bracket, and a simulated aero engine; and (**b**) shows the site layout for collecting video data. In addition, LED point light sources and colorful analog cables are arranged in the chamber, in order to simulate the real scene.

As far as we know, SAES is the first published data set for aero engine anomalous spark detection. The data set contains 26,382 images and correlated spark tags in total. The images come from a video of 17 min and 35 s in length. We used 361 Hikvision DS-2CD3T45FP1-LS cameras with a frame rate of 25 fps. All the images were taken from a simulation aero engine chamber, which we constructed. The inner chamber comprised a set of complex interferences, including complex illumination changes, colorful shaking cables, aero engine vibration, flickering flames, unsteady video interface, and shining metal surfaces. Figure 8 presents the data collection scene used for SAES.

SAES is a naturally distributed, realistic, and extremely challenging spark detection data set, which made it the best choice to test the performance of spark detection models. Specifically, the simulated aero engine anomalous spark data set had the following characteristics:

- Sparks:

We used an angle grinder to cut the metal, in order to produce high-speed sparks. There was no strict limit on the angle of cutting, the type of metal to be cut, and the intensity of cutting. The position of the metal being cut is shown on the side of the images. A wide range of shapes, colors, and flight paths of sparks were present in the scene.

- Data samples:

The data set included 26,382 images in total, along with spark labels for each image. Each image had a width of 856 and a height of 480. In practical applications, the frame rate of most cameras used to monitor aero engines was 25 frames per second. Therefore, all the images in the data set were taken from a video with a frame rate of 25 fps, and a length of 17 min and 35 s. Furthermore, the dataset was in YOLO format.

- Resolution:

The camera used to capture the data was a Hikvision DS-2CD3T45FP1-LS. The video encoding mode was set to H.265, the frame rate was set to 25 fps, and the resolution was set to 2560 × 1440. Considering realistic environments, the sharpness of the videos captured by the camera would be reduced under unavoidable factors. We used bilinear interpolation to reduce the resolution of each image captured by the camera to 856 × 480 in order to simulate a realistic situation.

**Figure 9:**
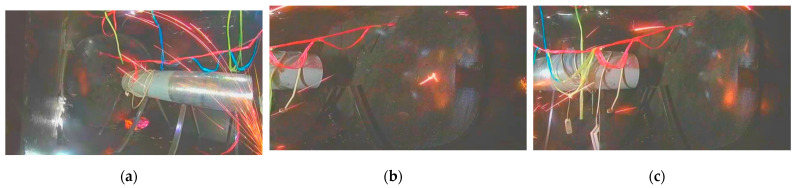
Some of the image data in SAES: (**a**) radiate sparks, flame interference, point light source interference, metal edge and metal reflection interference, and camera jitter interference; (**b**) scattered sparks; and (**c**) scattered sparks capture by a camera in another position.

- Application:

Although the spark data set had a large amount of data, the background, shapes, and colors of sparks, and the interference types included in the dataset were still limited from the perspective of model training. We attempted to use it to train our model, but it did not work very well; thus, we only used it for testing. The specific training method is introduced in the following section. Figure 9 shows some of the images in SAES.

### 3.2. Spark Data Set for Model Training

Due to the lack of aero engine spark data sets and the limited spark morphological features generated by the simulated aero engine, it was difficult to train a spark detection model with excellent performance. However, inspired by a raindrop appearance generation algorithm [39], we proposed a method to generate sparks by computer simulation.

First, 650 kinds of sparks with different patterns, such as ellipse and segment, were generated using the raindrop appearance modeling algorithm. This method took the influence of focal length, object distance, and raindrop velocity into account, which led to different spark morphologies, preserving the realistic features of raindrops. The raindrop appearance is simulated by:

(17) $$b(a,z)=a\frac{f}{z}$$

(18) $$s(a)=-0.2+5.0a-0.9a^{2}+0.1a^{3}$$

(19) $$l(a,z)=(a+s(a)e)\frac{f}{z}$$

(20) $$g(x,y;a,z,\theta ,\mu )=\int_{0}^{l(a,z)}\exp\left(-\frac{{\left(x-\cos(\theta )\gamma -\mu_{x}\right)}^{2}+{\left(y-\sin(\theta )\gamma -\mu_{y}\right)}^{2}}{b{\left(a,z\right)}^{2}}\right)d\gamma$$

where f is the focal length, z represents the object distance, a is the diameter of the object, and b denotes the width of the stripes captured from the high-speed moving raindrop. For a spark, its velocity s can be approximated by a. Further, l is the length of the raindrop, $\left(\mu_{x},\mu_{y}\right)$ denotes the image center, θ denotes the moving direction of the raindrop, (x,y) shows the raindrop position, and e represents exposure time.

In fact, the mean and standard deviations of the variables were closely related to the shape and size of the raindrops generated. The appearances of the streak of falling particles in the dynamics proposed by Foote and Magono could be approximated as a motion-blurred Gaussian.

The dynamics of falling particles were well-understood, and it was simple to determine the general shape of the streak that a given raindrop will create. Based on the shape, the streak’s appearance was then approximated as a motion-blurred Gaussian. Specifically, the image of a raindrop was approximated as a Gaussian, which appeared similar to a slightly out-of-focus sphere. As the particle moved in space, the image it created was a linear motion blurred version of the original Gaussian. If the sphere was larger or closer to the camera, the Gaussian would have had a higher variance. If it was falling faster, then it would be blurred into a longer streak.

It was believed that the sparks generated in the experimental chamber of the aero engine had high velocities. Therefore, even if the sparks did not fall vertically, their streak morphology was approximated as a motion-blurred Gaussian. In the meantime, unlike the velocity of the raindrop, which depended on the diameter of the raindrop, the velocity of the spark was a variable in the formula that generates the spark. Thus, the Gaussian function of spark appearance shape modeling is as follows:

(21) $$b(a,z)=a\frac{f}{z}$$

(22) $$l(a,z,s)=(a+s\cdot e)\frac{f}{z}$$

(23) $$g(x,y,a,z,s,\theta ,\mu )=\int_{0}^{l(a,z,s)}\exp\left(-\frac{{\left(x-\cos(\theta )\gamma -\mu_{x}\right)}^{2}+{\left(y-\sin(\theta )\gamma -\mu_{y}\right)}^{2}}{b{\left(a,z\right)}^{2}}\right)d\gamma$$

where f is the focal length, z represents the object distance, a is the diameter of the object, and b denotes the width of the stripes captured from the high-speed moving spark. s is the velocity of the spark. Further, l is the length of the spark, $\left(\mu_{x},\mu_{y}\right)$ denotes the image center, θ denotes the moving direction of the spark, and (x,y) shows the spark position. e represents exposure time.

The mean μ in the Gaussian function determined the position of the added spark in the image, and the velocity s and the standard deviation b in the Gaussian function jointly determine the shape of the spark. In addition, θ determined the angle of the spark showing in the image.

In the process of simulating sparks, θ and μ were randomly selected according to the uniform distribution to ensure that the generated sparks may appear at any position with any angle in the image. The standard deviation b in the Gaussian function depended on the size of the spark, the focal length of the camera, and the distance between the spark and the camera. These parameters and the velocity of the sparks were all determined by the size of the chamber of the aero engine and the engine running state.

After the sparks were simulated and generated, we grayed the generated images. According to the experimental observations, gray values were limited to the range of more than or equal to 100, and less than or equal to 255. The grayscale transformation formula is as follows:

(24) $$G^{*}(x)=100+\frac{G(x)}{255}\cdot (255-100)$$

**Figure 10:**
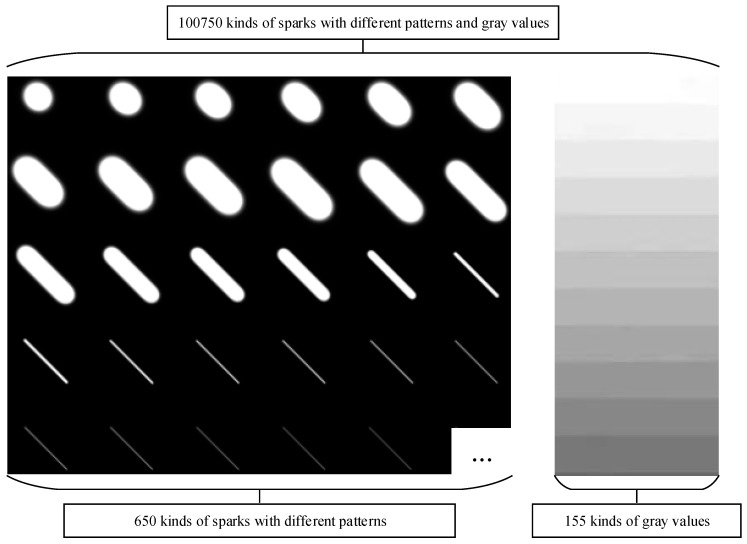
Gray images of sparks in 155 different gray values and 650 different shapes generated by the Gaussian function.

Figure 10 shows the gray images of simulated sparks.

To make the dataset more realistic, different forms of sparks were scaled to different ranges. Then, the sparks were rotated to augment the variety and authenticity of their patterns. Next, we embedded them into the normal aero engine video randomly and generated label information at the same time. It is worth noting that, when multiple sparks were gathered at the same position in an image, forming a cluster of sparks, the spark features at that position are complex, being inconsistent with the line-segment morphological features of a single spark. When generating the corresponding label information of sparks, 1–15 sparks should be included in a tag box, in order to simulate the morphological features of a cluster of sparks. Importantly, the used normal aero engine video included complex illumination changes, colorful shaking cables, aero engine vibration, flickering flames, unsteady video interface, and shining metal surfaces.

**Figure 11:**
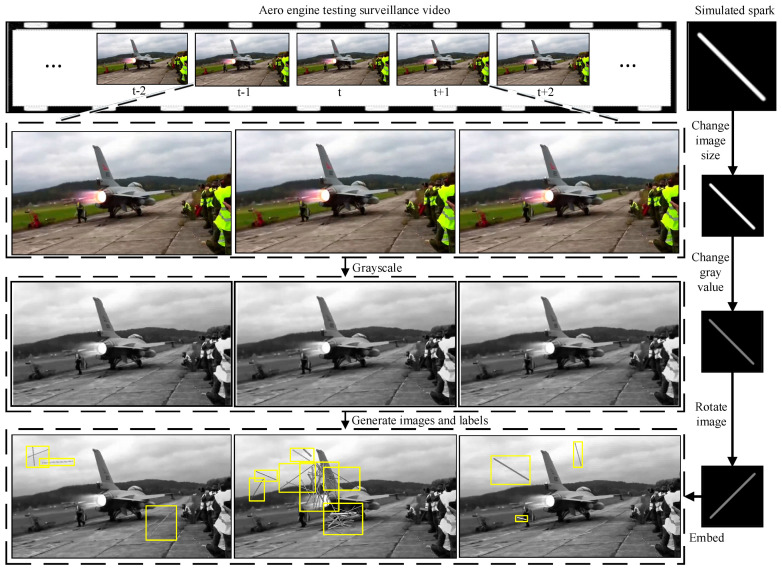
The schemaic digram of adding gray images of Gaussian-generated sparks to normal engine images and generating corresponding labels.

**Figure 12:**
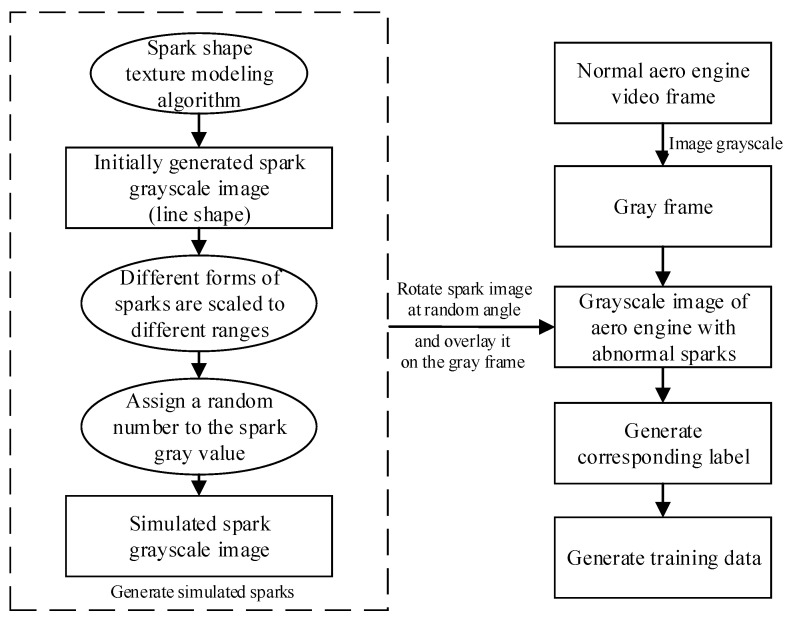
Flow chart of generating training data.

Generating the simulation data set using this method allowed for high-efficiency and rewarding deep learning model training. Figure 11 shows a schematic diagram of generating the simulated aero engine anomalous spark data set. In the next part, our experiments on the open-source anomalous spark data set show that a spark detection model with high precision and robustness can be trained using the data set generated by our proposed simulated spark generation method. The flow chart of generating the training data is shown in Figure 12.

### 3.3. Evaluation Metrics

**Figure 13:**
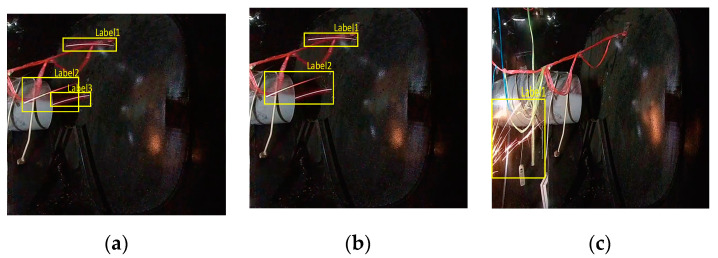
Images and visualization labels in the SAES data set: (**a**,**b**) two different correct labels corresponding to the same image, where the position of a single spark in the image can be correctly described by label 1 in (**a**) and in (**b**); however, the two close sparks corresponding to labels 2 and 3 in (**a**) can also be correctly described by label 2 in (**b**,**c**) A cluster of sparks and its corresponding label.

There are multiple possible reasonable labels for multiple sparks. As shown in Figure 13a, label 1 could accurately describe the location of a single spark in the image. However, the two sparks corresponding to labels 2 and 3 in Figure 13a are too close, such that label 2 in Figure 13b can also directly describe the positions of the two sparks. As shown in Figure 13c, this confusion happens more often for a cluster of sparks, which may correspond to many reasonable labels. Therefore, the AP was not suitable when evaluating the performance of spark detection models.

We expanded the true positive judgment rules in the object detection AP calculation in order to ensure that the AP was more appropriate to evaluate the performance of SAVSDN. In addition to what was deemed as true positives by the original rules, we believe that, if the prediction box satisfies two conditions at the same time, it can also be considered as a true positive. The first condition is that the center point of the prediction box must be located in any label box, while the other condition is that the ratio of the area of the prediction box and of the area of every label box in the area of the prediction box must be within a reasonable range r. Considering that the prediction box may be located inside or outside the label box, we used r=[0.5,1.5]. We used this variant of AP to evaluate the performance of SAVSDN. Next, we set the threshold value for the parameter intersection over union (IoU) of the origin AP as 0.5.

**Figure 14:**
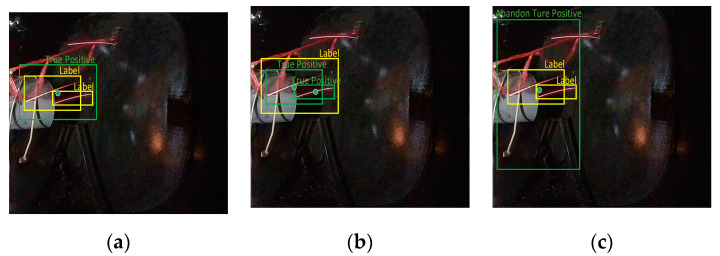
(**a**,**b**) contain two true positives identified by the additional rules, while (**c**) contains an abandoned true positive, as identified by additional rules. In (**a**), the green prediction box is located outside the yellow label box; in (**b**), the green prediction box is located inside the yellow label box; and, in (**c**), the green prediction box outside the yellow label box is so large and inaccurate that it is considered to be an abandoned true positive.

The green prediction boxes in Figure 14a,b were judged as true positives, according to the additional judgment rules. Although the center point of the green prediction box in Figure 14c was located in the label box, the ratio of the area of the prediction box and all areas of the label boxes, which are in the area of the prediction box, exceeded the reasonable range. We can also easily see that this prediction box was too large and, thus, inaccurate in describing the location of the sparks; thus it was considered an abandoned true positive.

In addition, we also used specific precision, recall, and accuracy, in order to evaluate the timeliness and accuracy of the system alarms. We regarded images with one or more sparks as positive samples, while the other images were regarded as negative samples. When the input of the model is a positive sample and the model detects any spark in the image, the prediction is a true positive (TP); otherwise, it is a false negative (FN). When the input of the model is a negative sample and the model detects any spark in the image, the prediction is a false positive (FP); otherwise, it is a true negative (TN). The specific precision, recall, and accuracy are calculated as follows:

(25) precision=TP/(TP+FP)

(26) recall=TP/(TP+FN)

(27) accuracy=(TP+TN)/(TP+FP+FN+TN)

### 3.4. Implementation Details and Ablation Experiments

To understand the elements in the cascaded model and the effectiveness of the training data set generation method, we conducted a number of ablation studies based on our open-source simulated aero engine spark data set. The generated training data set was used to train our model, while the SAES data set we proposed was used as the test data set. In fact, the images of SAES were collected in the experimental chamber of our simulated aero engine using a 361 Hikvision DS-2CD3T45FP1-LS camera. The sparks were generated by using an angle grinder cutting metal rather than Gaussian function. SAES is a naturally distributed, realistic and extremely challenging spark detection data set, which made it the best choice to test the performance of spark detection models. In addition, we have tried to train our model with real spark data which led to poor training results due to the singularity of shape, velocity, color, and background of sparks. Considering the existence of a large number of normal aero engine test images and the Gaussian function that can generate simulated spark images, we used the Gaussian function and a large number of normal aero engine test images to create a training data set to train the model.

For the environment settings, we first built a deep learning experimental model based on PyTorch, including the proposed codec model and benchmark models for spark detection, which were also based on PyTorch. All of the experiments were performed on a PC server, with an Intel CPU 8700K 5.0 GHz, NVIDIA RTX2080Ti 12 GB, and 16 GB of RAM. The benchmark models used the same hyperparameters as our proposed model. The batch size was 8, we considered 100 epochs, and the learning rate was 0.001. The ADAM function was used as the training optimizer for all deep learning models. All models output the types and positions of the targets by the object detectors.

Comparison of the 11 benchmark models and SAVSDN:

- ConvLSTM YOLOv5_09: the classical ConvLSTM was directly used to encode the video image sequences with a length of nine frames, and the coding results were sent into YOLOv5 to output the spark detection results for the whole nine frames;
- ConvLSTM YOLOv5_06: the structure was the same as ConvLSTM YOLOv5_09, but YOLOv5 only output the spark detection results for the latter six frames;
- ConvLSTM YOLOv5_03: the structure was the same as ConvLSTM YOLOv5_09, but YOLOv5 only output the spark detection results for the latter three frames;
- SEQ2SEQ YOLOv5_09: the classical ConvLSTM-based sequence-to-sequence deep learning network was used to encode the video image sequences with a length of nine frames. Then, another ConvLSTM decoded the spatio-temporal context vector to obtain the deep feature image, which was then sent to YOLOv5 to output the spark detection results for the whole nine frames;
- SEQ2SEQ YOLOv5_06: the structure was the same as SEQ2SEQ YOLOv5_09, but YOLOv5 only output the spark detection results for the last six frames;
- SEQ2SEQ YOLOv5_03: the structure was the same as SEQ2SEQ YOLOv5_09, but YOLOv5 only output the spark detection results for the last three frames;
- SAVSDN_non-motion-sensing encoder: the structure was similar to the proposed spark detection model. The ConvLSTM decoder was retained, while the initial hidden state was replaced with the results of encoding the first seven frames directly by ConvLSTM;
- SAVSDN_non-parameter-sharing motion-sensing encoder: the structure was similar to the proposed spark detection model. Four independent ConvLSTMs were used to replace the ConvLSTM-based parameter-sharing motion-sensing encoder, in order to encode the four groups of image sub-sequences, respectively;
- SAVSDN_motion-sensing encoder_ht: the structure was similar to the proposed spark detection model, but ht was set as the output of the motion-sensing encoder.
- SAVSDN_interference bright line segments encoder_h_t_: the structure was similar to the proposed spark detection model, but h_t_ was set as the output of the interference bright line segments encoder;
- SAVSDN_decoder_c_t_: the structure was similar to the proposed spark detection model, but c_t_ was set as the output of the decoder;
- SAVSDN: Our full model.

The results of the ablation studies are shown in Table 1. We used classic sequence-to-sequence codecs and compared them with the codecs of our model; the detector cascaded by these codecs was YOLOv5.

ConvLSTM YOLOv5_09 and SEQ2SEQ YOLOv5_09 were both trained using all the sparks in the input image sequences with a length of nine frames. In this experiment, ConvLSTM YOLOv5_09 was generated by removing the ConvLSTM-based decoder in SEQ2SEQ YOLOv5_09. The AP increased from 11% to 17%, which indicates that directly transmitting the result of encoding spatio-temporal sequences based on ConvLSTM to the object detector is not a good idea, while the use of the decoder can improve the results of the encoders and, thus, the AP.

Considering the real-time and lightweight requirements of aero engine spark detection, we did not apply the time-consuming and complex attention mechanism to our model to solve the problem that the features extracted by ConvLSTM are diluted by subsequent inputs. It is obvious that the shorter the segments of the image sequences to be detected, the fewer spark features that need to be memorized by the long-term state of ConvLSTM, and the less likely it is that the extracted spark features are diluted by the subsequent inputs. The network structures of ConvLSTM YOLOv5_09, ConvLSTM YOLOv5_06, and ConvLSTM YOLOv5_03 were identical. The difference between them was the segments of the image sequences to be detected. The experiment results were consistent with our expectations. Compared with ConvLSTM YOLOv5_09, the AP of ConvLSTM YOLOv5_03 increased from 11% to 19%, while the performance of ConvLSTM YOLOv5_06 lay between them. The experimental results of SEQ2SEQ YOLOv5_09, SEQ2SEQ YOLOv5_06, and SEQ2SEQ YOLOv5_03 also verified this point. The ablation experiment showed that we can shorten the length of the image sequence required by the network to detect sparks, thus reducing the dilution of the extracted features and improving the spark detection performance. The proposed information fusion mechanism was also inspired by the results of this ablation experiment.

The information fusion mechanism was used to fuse the initial long-term hidden state with the input and then decode it with the ConvLSTM-based decoder. The long-term hidden state of the encoder for the interference bright line segments and the motion-sensing encoder serves as the initial long-term hidden state and the input to the decoder, respectively. The short-term state of the decoder is the output. In the ablation studies, SAVSDN_motion-sensing encoder_ht, the Spark detection model for the interference bright line segments encoder_ht, the Spark detection model_decoder_ct, and our spark detection model had a similar network structure, only changing the signal categories transmitted between ConvLSTMs. Compared to the 83% AP achieved by our spark detection model, the APs of the former three models were 43%, 35%, and 55%, respectively, showing significant decreases. This indicates that the information fusion mechanism based on ConvLSTM requires a correct network structure to ensure that the necessary spatio-temporal features are extracted from the image spatio-temporal sequences. The other two models of the information fusion mechanism—the spark detection model for the non-motion-sensing encoder, and the spark detection model for the non-parameter-sharing motion-sensing encoder—did not extract the same spatio-temporal features from the first seven frames and the last three frames. The former directly encodes the first seven using ConvLSTM and had an AP of 62%. The latter uses a parameter-independent ConvLSTM-based motion-sensing encoder and had an AP of 71%. Compared with our spark detection model, both of them showed significant performance degradation. This experiment demonstrated that, when the ConvLSTM-based decoder was used as the information fusion device, two kinds of feature information—the global scene features, as well as the long-term features extracted from the first seven frames and the correlated non-linear spatio-temporal features from the last three frames—are needed to ensure the consistency of the extracted features, in terms of the decoding quality.

### 3.5. System Comparison

We conducted a comparison of three advanced image object detectors, two advanced video object detectors, and SAVADN. Table 2 and Table 3 show the results of the comparison, using three state-of-the-art image object detectors (RetinaNet [43], YOLOv4 [44], and YOLOv5) and two state-of-the-art video object detectors (LSTS [34] and MEGA [32]). It can be seen that our algorithm significantly outperformed the other algorithms.

On our SAES data set, we compared our method with the five other advanced methods. RetinaNet, which achieved a COCO test-dev AP of 39.1%, had only 2% accuracy on our data set. Another image object detector, YOLOv5, had an AP of only 5% after training on our spark data set, and had high over-detection and error-detection rates due to the background interference. We found that the video object detector, MEGA, does not apply to spark detection, as MEGA uses the information of candidate boxes in adjacent frames to detect objects in the current frame, as did LSTS. Current video target detectors successfully detect occluded objects in the video, but are ineffective for fast-moving line objects, such as sparks. The MEGA and LSTS algorithms had APs of only 7% and 4%, respectively, on our spark data set. In addition, compared with the above algorithms, the proposed algorithm had a faster processing time and could process video in real-time. One thing to note is that, due to the particularity of the model structure, our codec-concatenated target detector YOLOv5 can output three pictures at a time. Therefore, although our codec has some time delay, the average processing speed of our model for each picture is faster than that of ordinary YOLOv5. These comparison results validate two of our innovations: the cascading codec-object detector structure and the information fusion mechanism. Based on our open-source data set of simulated aero engine anomalous sparks, by applying the cascading codec-object detector structure, the AP increased surprisingly to 83% from 5% and 7%, respectively, when compared to YOLOv5 and MEGA.

As mentioned in Section 3.3, we designed specific precision, recall, and accuracy metrics to describe the spark alarm accuracy of our model. The greater the precision, the less over-detection. Furthermore, the larger the recall, the fewer missed detections. By comparing our algorithm with the other five benchmark algorithms detailed above, considering the precision, recall, and accuracy values, we found that our algorithm had a lower over-detection rate than the image object detectors and a lower misdetection rate than the video object detectors.

### 3.6. Visualization Results

**Figure 15:**
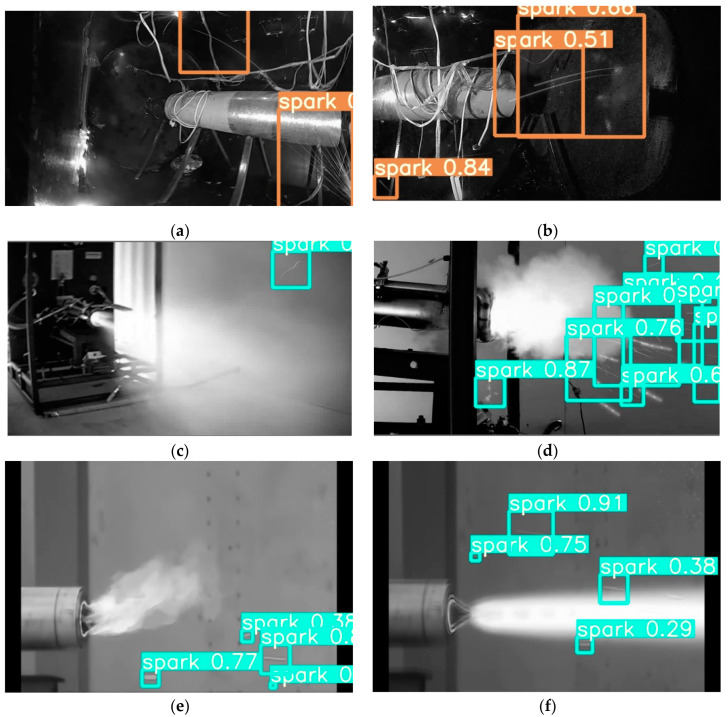
The SAVSDN spark detection results in different images. Each of the boxes is a result of the model, where the words on each box represent the category and the numbers show the confidence level. (**a**,**b**) demonstrate the detection result of SAVSDN on the SEAS data set; (**c**–**f**) show the detection results of SAVSDN on other engines.

The spark detection results of SAVSDN are shown in Figure 15.

To better understand the information fusion mechanism of the codec, we visualized the intermediate state matrices of each ConvLSTM in the codec, which reflect the temporal dependency features and the spatio-temporal non-linear correlation features.

**Figure 16:**
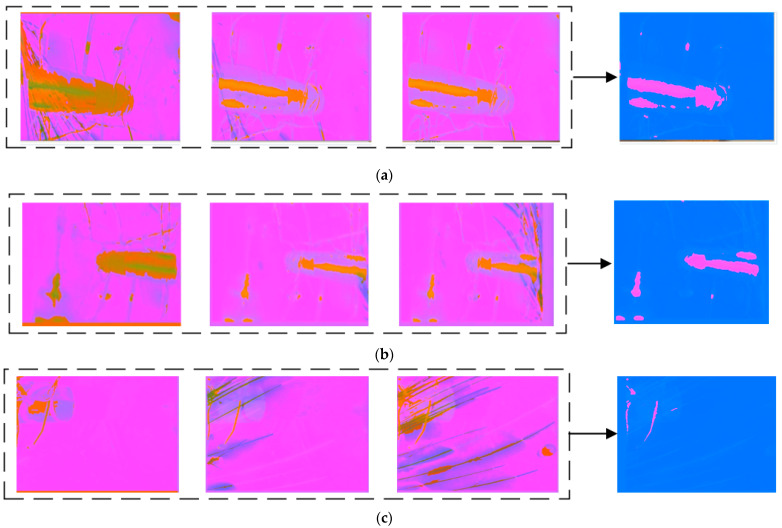
(**a**–**c**) present the encoding visualization results, generated from input image sequences with various anomalous sparks by the ConvLSTM-based motion-sensing encoder and ConvLSTM-based interference encoder. The visualization method scales each value of the three-channel two-dimensional tensors, output by the activation function, in the interval from 0 to 255, and displays it as an RGB image.

As shown in Figure 16, the visualized images in the left dashed boxes are the short-term spatio-temporal feature images of 1st–3rd frames, 3rd–5th frames, and 5th–7th frames extracted by the parameter-sharing motion-sensing encoder. The images on the right side are the long-term feature images, extracted from the left images by the encoder for the interference bright line segments. It can be seen that the long-term feature images only contain the interference from the vibrating aero engine, the colorful cables, and the vibrating aero engine bracket, but no spark features, demonstrating that the special structure of the encoder can exploit the spatio-temporal features of the long-term interference in the scene.

**Figure 17:**
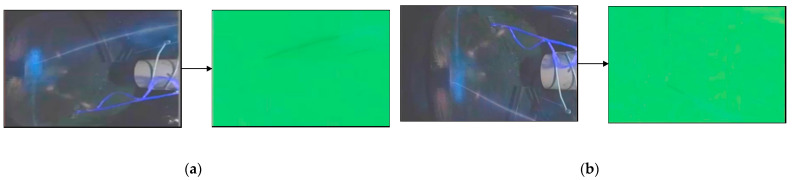

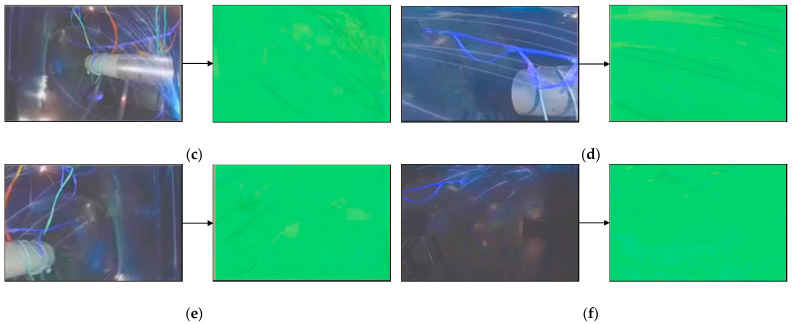
(**a**–**f**) show the visualization results of aero engine images with anomalous sparks, generated by the encoder and decoder at six different moments. The visualization method scales each value of the three-channel two-dimensional tensors, output by the activation function, into the interval from 0 to 255, and displays it as an RGB image. The deep feature images only include the spark texture features.

The image on the left of Figure 17 is the superimposed image of the last three input frames. Specifically, we added the three-channel brightness values of the three images and divided them by three to observe the sparks in the last three frames conveniently. The images on the right side are the deep feature images generated by encoding and decoding. Importantly, we found that only the spark features were included in the deep feature images, which indicates that the information fusion mechanism based on the ConvLSTM decoder successfully integrated the long-term spatio-temporal feature images of the interference and the short-term spatio-temporal feature images. Thus, we can easily extract the spark non-linear correlation features, demonstrating that our model can perceive sparks and bright line segments in the scene, and can effectively distinguish sparks from the interference the bright line segments. Figure 17 shows the visualization results.

## 4. Conclusions

To solve the key problems in the process of detecting anomalous sparks in aero engine testing, we proposed a sequence-to-sequence deep learning framework based on an information fusion mechanism. A method for generating training data was also proposed. According to the features of sparks in the spatio-temporal domain, the proposed model integrates a deep learning structure for the sequence-to-sequence codec, utilizing the ConvLSTM network. The core framework of the model was analyzed and discussed in detail.

Moreover, we published a challenging open-source data set, named SAES, to test the performance of the proposed algorithm, SAVSDN. Ablation experiments were carried out to demonstrate that, compared with five state-of-the-art image object detection and video object detection algorithms, our model could outperform all of them. Specifically, the proposed video codec in SAVSDN is an improvement of the classic sequence-to-sequence model, which increases the specific AP index from 23% to 83%. However, for advanced image object detectors such as Retina Net, YOLOv4 and YOLOv5, and advanced video object detectors such as LSTS and Mega, the specific AP indexes are only 2%, 9%, 5%, 4%, and 7%. As for the accuracy of system alarms, the SAVSDN has a precision of 77% and a detection speed of 7.1 ms per frame. The main advantage of SAVSDN is the low ratio of over-detection. The spark detection precision of RetinaNet, YOLOv4, YOLOv5, LSTS, MEGA, and SAVSDN are 3%, 4%, 3%, 4%, 2% and 63%, respectively. Their recall ratios are 85%, 79%, 82%, 12%, 16% and 92%, respectively. Therefore, SAVSDN is far more superior to the five advanced object detectors in these five metrics. It was verified that the model can exploit and learn the non-linear correlation deep features extracted from the spatio-temporal data, which can be used to distinguish sparks from interference bright line segments. Under the premise of ensuring real-time performance, the over-detection and misdetection of anomalous sparks were reduced.

In the future, our main line of study will be to collect realistic spark images during the aero engine testing to further improve and expand the newly published data set. Further research and improvement will also be carried out in order to realize the effective intelligent detection of anomalous sparks in aero engine tests. Eventually, we believe that the proposed method can serve to liberate human resources and promote the development of aero engine technology.

## Figures and Tables

**Table 1:** Ablation studies and the corresponding performance for spark detection.

Method	AP
ConvLSTM YOLOv5_09	11%
ConvLSTM YOLOv5_06	16%
ConvLSTM YOLOv5_03	19%
SEQ2SEQ YOLOv5_09	17%
SEQ2SEQ YOLOv5_06	21%
SEQ2SEQ YOLOv5_03	23%
SAVSDN_non-motion-sensing encoder	62%
SAVSDN_non-parameter-sharing motion-sensing encoder	71%
SAVSDN_motion-sensing encoder_h_t_	43%
SAVSDN_interference bright line segments encoder_h_t_	35%
SAVSDN_decoder_c_t_	55%
SAVSDN	83%

**Table 2:** System comparison and the corresponding performances for spark detection.

Method	AP	Runtime
RetinaNet	2%	198.3 ms
YOLOv4	9%	28.1 ms
YOLOv5	5%	8.5 ms
LSTS	4%	45.6 ms
MEGA	7%	120.6 ms
SAVSDN	83%	7.1 ms

**Table 3:** System comparison and the corresponding performances for spark warning.

Method	Precision	Recall	Accuracy
RetinaNet	3%	85%	7%
YOLOv4	4%	79%	9%
YOLOv5	3%	82%	8%
LSTS	4%	12%	2%
MEGA	2%	16%	1%
SAVSDN	63%	92%	77%

## Data Availability

Our code, dataset, and detection results will be made publicly available at github.com/koujie317099295/SAVSDN-SAES-SAVSDN_detection_result (accessed on 26 June 2021).
